# PARTIAL RETRACTION: Ethics, COVID-19 and nursing vulnerability: analysis of photographs released by the media

**DOI:** 10.1590/0034-7167.e20267901retr

**Published:** 2026-08-31

The editorial team of the Brazilian Journal of Nursing decided, after analysis, to retract Figure 5 from the following original article:

Sena GP, Fontenele AL, Duarte ADCA, Ferreira GI, Guilhem DB. Ethics, COVID-19 and nursing vulnerability: analysis of photographs released by the media. Rev Bras Enferm. 2022;75(Suppl 2):e20210960. https://doi.org/10.1590/0034-7167-2021-0960

This was requested by the patients depicted. We regret any misunderstanding caused to our readers.

**Dulce Aparecida Barbosa**

Editor in Chief

