# Supplementary material for: PARTIAL RETRACTION: Ethics, COVID-19 and nursing vulnerability: analysis of photographs released by the media
Source: Rev Bras Enferm. 2026 Aug 31;79:e20267901retr. doi: 10.1590/0034-7167.e20267901retr (PMC13543776; doi:10.1590/0034-7167.e20267901retr)
Supplement: Related texts

# Retraction: RETRATAÇÃO PARCIAL: Ética, COVID-19 e vulnerabilidade da enfermagem: análise de fotografias divulgadas pela mídia

**DOI:** 10.1590/0034-7167.e20267901retrpt

A equipe editorial da Revista Brasileira de Enfermagem decidiu, após análise, retratar a figura 5 do seguinte artigo original:

Sena GP, Fontenele AL, Duarte ADCA, Ferreira GI, Guilhem DB. Ethics, COVID-19 and nursing vulnerability: analysis of photographs released by the media. Rev Bras Enferm. 2022;75(Suppl 2):e20210960. https://doi.org/10.1590/0034-7167-2021-0960pt

Trata-se de solicitação das pacientes retratadas. Lamentamos qualquer mal-entendido causado ao nosso leitor.

**Dulce Aparecida Barbosa**

Editora Chefe
